# The association between lifestyle-related risk factors and survival in patients with colorectal cancer in an urban South African cohort

**DOI:** 10.4314/ahs.v22i1.38

**Published:** 2022-03

**Authors:** Megan Whelan, Heleen van Aswegen, Ronel Roos, June Fabian, Brendan Bebington

**Affiliations:** 1 Department of Physiotherapy, School of Therapeutic Sciences, Faculty of Health Sciences, University of the Witwatersrand, South Africa; 2 Wits Donald Gordon Medical Centre, University of the Witwatersrand, Johannesburg, South Africa1; and; 3 Department of Internal Medicine, Faculty of Health Sciences, University of the Witwatersrand, Johannesburg, South Africa; 4 Department of Surgery, School of Clinical Medicine, Faculty of Health Sciences, University of Witwatersrand, Johannesburg, South Africa; 5 Colorectal Unit, Wits Donald Gordon Medical Centre, Johannesburg, South Africa

**Keywords:** Cancer survival, risk factors, physiotherapy

## Abstract

**Background:**

Lifestyle-related factors have been linked with risk for colorectal cancer. Data describing the relationship between lifestyle factors of South African patients who present with colorectal cancer and their survival is sparse.

**Objectives:**

The objectives were to describe the profile of patients with colorectal cancer; to determine the association between lifestyle-related factors and survival, and to compare results of patients in the private and public sectors.

**Methods:**

A retrospective review and secondary analysis of information of patients with colorectal cancer were conducted. The independent samples t-test and Mann Whitney U test were administered to determine differences in the clinical presentation. Pearson's Chi-Squared and Eta (η) tests were used to determine the association between survival and lifestyle-related factors.

**Results:**

Data of 441 patients were included. When compared to the public sector cohort, patients in the private sector cohort were older (p=0.0110), had earlier stages of cancer at the time of diagnosis (p<0.001), had a higher percentage of current alcohol consumption (p<0.001) and had higher survival rates (p<0.001). Waist circumference was shown to have a large-strength effect on survival (η2=0.266).

**Conclusion:**

Emphasis should be placed on anthropometric screening and education to effect long-term behaviour change. Physiotherapists are well placed to provide screening and non-pharmacological interventions for patients with colorectal cancer.

## Introduction

In South Africa, colorectal cancer is the fourth most prevalent type of cancer^1^. According to the 2014 National Cancer Registry, the crude incidence of colorectal cancer for men and women in South Africa is 7.34/100 000 and 5.86/100 000 respectively^2^.

Colorectal carcinogenesis involves several complex biological pathways^3^. Lifestyle-related risk factors include cigarette smoking, heavy alcohol consumption, nutrition-related practices, obesity, and lack of physical activity^4^,^5^. Furthermore, research shows that physical inactivity, body mass index (BMI), and smoking may influence survival after a colorectal cancer diagnosis^6^,^7^.

Smoking and alcohol consumption are both associated with colorectal cancer. Tobacco carcinogens may damage or alter the expression of important cancer-related genes^8^. The carcinogens in tobacco have also been linked with the development and growth of adenomatous polyps, the precursor lesions for colorectal cancer^9^. There are several possible biological mechanisms to explain the higher mortality rates in individuals who smoke at the time of and following the diagnosis of colorectal cancer. Smoking may result in impaired tobacco carcinogen detoxification which could promote residual tumour cell growth either by angiogenesis promotion or by chemotherapy resistance^10^. Secondly, smoking may contribute to abnormal promoter methylation which results in regulatory gene silencing in tumour progression^11^. Relating to alcohol, there are several mechanisms of alcohol-associated carcinogenesis including nutritional deficienies, modulation of cellular regeneration, and the carcinogenic effects of acetaldehyde which is the main metabolite of ethanol^12^. Long term alcohol consumption induces cytochrome P-4502E1 in the liver and gastrointestinal mucosal cells, which increases reactive oxidative species generation, leading to activation of various carcinogens - similar to those in cigarette smoke^12^. Even low daily doses of alcohol can enhance carcinogenesis^12^. Smoking, but not alcohol consumption, has been associated with an increased risk of mortality following colorectal cancer diagnosis^7^,^13^.

The relationship between BMI and colorectal cancer outcomes is complex^14^,^15^. Central and general obesity have been shown to have a dose-dependent relationship with risk for colorectal cancer^16^,^17^. Overweight individuals with colorectal cancer have shown better overall survival outcomes^7^,^14^,^18^.

Evidence suggests that physical activity exerts an independent effect on risk for colorectal cancer^5^,^19^. Physical activity increases gut motility which in turn reduces faecal transit time^20^. Beyond risk, physical activity has also been linked with survival in patients with colorectal cancer^7^,^21^,^22^. Results of a large-scale European prospective study showed that prediagnosis leisure time activity was associated with improved survival in patients with colorectal cancer^22^. Physical activity may reduce tissue insulin and insulin-like growth factor levels as well as play a role in anti-inflammatory actions and immune modulation^23^. Activity-induced body changes may increase cancer treatment efficacy and could support counteracting cancer progression^22^.

The objectives of this study were to describe the profile of patients with colorectal cancer; to determine the association between lifestyle-related factors and survival; and to compare results of patients presenting with colorectal cancer at private sector hospitals to those presenting at public sector hospitals within a University teaching complex.

To our knowledge, data describing the relationship between lifestyle-related risk factors of South African patients who present with colorectal cancer and their survival is sparse. This information is vital to determine the need for management of modifiable risk factors in this patient population. As far as we know, this is the first study of this nature in a South African group of patients with colorectal cancer.

## Methods

Approval to conduct this study was obtained from the University of the Witwatersrand Human Research Ethics (Medical) committee (M181075). A retrospective review and secondary analysis of information captured on Research Electronic Data Capture (REDCap) hosted at the University of the Witwatersrand were conducted^24^,^25^.

### Patient sample and database information

The database includes patient information collected from the study sites based in the Academic Teaching Complex of the University of the Witwatersrand in Johannesburg. These sites included one private hospital (private university referral centre) and three public hospitals (two of which are tertiary referral centres and one is a secondary care facility)^26^. Inclusion criteria comprised patients 18 years or older, a confirmed histological diagnosis of primary colon or rectal adenocarcinoma, diagnosed within the last 12 months, and written informed consent. The records of a convenience sample of patients enrolled between 1 January 2016 and 30 June 2018, with at least six months follow-up data were included.

Information was collected for the following variables: demographics (age, gender, and self-reported race), anthropometrics (weight, height, and waist circumference), lifestyle factors (physical activity, smoking, and alcohol use) and cancer staging. Outcome was reported as survival - disease or disease-free. Overall survival was determined from the date of recruitment to date of death or date of last contact session.

Three trained data capturers entered data onto the REDCap system. Patients were referred by specialists and from relevant departments such as chemotherapy, radiotherapy, and from multidisciplinary meetings hosted at the various study sites. At the time of data capturing, specialists were available to answer any questions and queries regarding the data. The data capturers assisted with data extraction from REDCap onto excel spreadsheets.

### Outcome measures

The staging of cancer was measured using the American Joint Committee on Cancer (AJCC) Tumor-Node-Metastasis (TNM) staging model^27^. The AJCC tool (7th edition) categorises the malignancy from stage 0 (presence of a primary tumour) to stage IVB (distant metastases in more than one site)^28^. The tool demonstrates good prognostic validity^28^.

Physical performance was measured using the Eastern Cooperative Oncology Group Scale of Performance Status (ECOG)29. The ECOG is a scale that measures patients' functional status including self-care ability and daily activity. The scale was designed to measure the impact of a patient's disease on their ability to perform various activities of daily living and was created specifically to be used in the field of cancer research. The ECOG score is often used to prognosticate for outcomes following cancer treatment. The scale grades patients according to their abilities (grade 0 - patients who are fully active and have no restrictions; grade 5 - patients who have died)^30^. The scale is known for its intraobserver reliability and simplicity^31^.

The Global Physical Activity Questionnaire (GPAQ) was used to measure physical activity. The questionnaire was designed to collect information on physical activity participation across three domains namely work activity, travel to and from work, and recreation32. The World Health Organisation (WHO) recommends that an individual should achieve 150 minutes of weekly moderate-intensity aerobic physical activity or 75 minutes of weekly vigorous-intensity aerobic physical activity or 600 met-minutes of combined weekly moderate-and-vigorous-intensity physical activity^33^. The GPAQ scoring is based on these recommendations and is a reliable and valid measure of changes in moderate-to-vigorous physical activity 34,35. Data was not collected for one sub-domain which resulted in incomplete overall GPAQ scores. This resulted in complete GPAQ data being available only for vigorous-intensity physical activity.

### Data analysis

Data obtained were analysed using IBM SPSS (version 25) software^36^. Data describing the profiles and clinical presentations of patients with colorectal cancer were summarised using descriptive analysis and reported as frequencies (%), means and standard deviation (SD) and median and interquartile range (IQR). The normality of distribution of continuous data was measured using the Shapiro Wilk test. The independent samples t-test and Mann Whitney U test were administered to determine differences in the presentation of those presenting at private versus public sector hospitals. The Kaplan-Meier method was used to plot survival data and the log rank test was used to compare survival between the two groups.

Pearson's Chi-Squared test was used to determine the association between survival and nominal variables (smoking and alcohol consumption). The strength of association was measured using the Cramer's V test: a value of 0 indicated no relationship existed, 0.05–0.10 represented a weak relationship, 0.10–0.15 represented a moderate relationship, 0.15–0.25 represented a strong relationship, and >0.25 suggested a very strong relationship^37^. Eta (η) test was administered to determine the associationetween survival and ratio variables (BMI, waist circumference, and vigorous-intensity weekly minutes). Eta-squared (η2) was used to determine the effect size. The following guidelines were used to interpret the strength of association for η2: 0.02–0.13 represented a small effect size, 0.13–0.26 represented a medium effect size, and >0.26 represented a large effect size38. The significance of findings was set at an alpha level of ≤0.05. Missing data that couldn't be recovered was coded and recorded as ‘missing’.

## Results

Overall, 441 patients met the eligibility criteria for inclusion in the study sample. Of those recruited, 152 (34.5%) were in the private sector and 289 (65.5%) were in the public sector.

### Demographic profile

The profile of this cohort is summarised in Table 1.

**Table 1:** Demographic profile of South African urban cohort presenting with colorectal cancer

	Private sector cohort (n=152)	Public sector cohort (n=289)	p-value
**Gender** Male Female	77 (50.7) 75 (49.3)	147 (50.9) 142 (49.1)	0.967
**Age** (yrs)	60 (51–67.75)	56 (46–65)	0.011
**Self-reported race** Caucasian Black Mixed race Indian East Asian Other	98 (64.5) 26 (17.1) 4 (2.6) 23 (15.1) 0 (0) 1 (0.7)	41 (14.2) 212 (73.4) 25 (8.7) 9 (3.1) 2 (0.7) 0 (0)	<0.001 (overall)

Gender (n, %), age (median, IQR), self-reported race (n, %).

IQR (interquartile range), n (number), yrs (years).

The term ‘other’ refers to patients that felt that their race did not fall under any of the given options.

Those with colorectal cancer in the private sector were significantly older (p=0.011) and predominantly from the Caucasian population group (p<0.001) when compared to the profile of patients with colorectal cancer from state hospitals.

### Cancer staging

The stage of cancer for both groups is represented in Table 2.

**Table 2:** Staging of colorectal cancer of South African urban cohort using AJCC

	Private sector cohort (n=152)	Public sector cohort (n=289)	p-value
**AJCC** Stage 1 Stage IIa Stage IIb Stage IIc Stage IIIa Stage IIIb Stage IIIc Stage IVa Stage IVb Missing	17 (12.4) 32 (23.4) 5 (3.6) 2 (1.5) 2 (1.5) 31 (22.6) 16 (11.7) 25 (18.2) 7 (5.1) 15	11 (4.6) 25 (10.4) 8 (3.3) 7 (2.9) 1 (0.4) 29 (12) 59 (24.5) 57 (23.7) 44 (18.3) 48	<0.001 (overall)

AJCC (n, %)

The cancer staging data are distributed in a bimodal manner. The majority of patients in the private cohort presented with Stage IIa and Stage IIIb colorectal cancer according to the AJCC whereas the greatest percentage of patients in the public cohort presented with Stage IIIc and Stage IVa colorectal cancer. The difference between the two groups was significant (p<0.001).

### Lifestyle profile

The ECOG scores (physical performance), modified GPAQ scores (physical activity), anthropometric measures, smoking, and alcohol consumption of both cohorts are summarized in Table 3.

**Table 3:** Anthropometric and lifestyle profiles of the study cohort

	Private sector cohort (n=152)	Public sector cohort (n=289)	p-value
**Anthropometric data** Weight (kg) Height (m) Waist circumference (cm) BMI (kg/m^2^)	65 (56.4–79) 1.67 (1.59–1.73) 89.5 (83–99) 24 (21–28)	67.9 (56–80.63) 1.65 (1.58–1.73) 92 (85–102) 25 (21–29)	0.364 0.567 0.108 0.212
**Alcohol consumption** Current alcohol consumer Previous alcohol consumer Never consumed alcohol Missing	86 (57) 19 (12.6) 46 (30.5) 1	83 (28.7) 84 (29.1) 122 (42.2) 0	<0.001 (overall)
**Smoking** Current smoker Previous smoker Never smoked	16 (10.5) 52 (34.2) 84 (55.3)	45 (15.6) 65 (22.5) 179 (61.9)	0.021 (overall)
**ECOG score** Grade 0 Grade 1 Grade 2 Grade 3 Grade 4 Missing	36 (30.5) 47 (39.8) 23 (19.5) 9 (7.6) 3 (2.5) 34	73 (35.3) 84 (40.6) 31 (15) 18 (8.7) 1 (0.5) 82	0.341 (overall)
**GPAQ score** Vigorous-intensity physical activity weekly minutes Vigorous-intensity minutes achieved Yes No Missing (n)	67.5 (193.6) 22 (19) 94 (81) 36	92.38 (387.9) 26 (12.5) 182 (87.5) 81	0.191 0.116

Anthropometric data (median, IQR), alcohol consumption (n, %), smoking (n, %), ECOG score (n, %), GPAQ score vigorous-intensity physical activity weekly minutes (mean, SD), vigorous-intensity minutes achieved (n, %).

SD (standard deviation), kg (kilogram), m (meters), m^2^ (meters squared), cm (centimetres).

There was no significant difference in anthropometric profiles between the two groups. There was a significant difference in smoking (p=0.021) and alcohol consumption (p<0.001) between the private and public sector cohorts. The largest percentage of patients in the private and public cohorts had never smoked whereas more than half of the private cohort were current alcohol consumers.

### Survival

**Figure 1:**
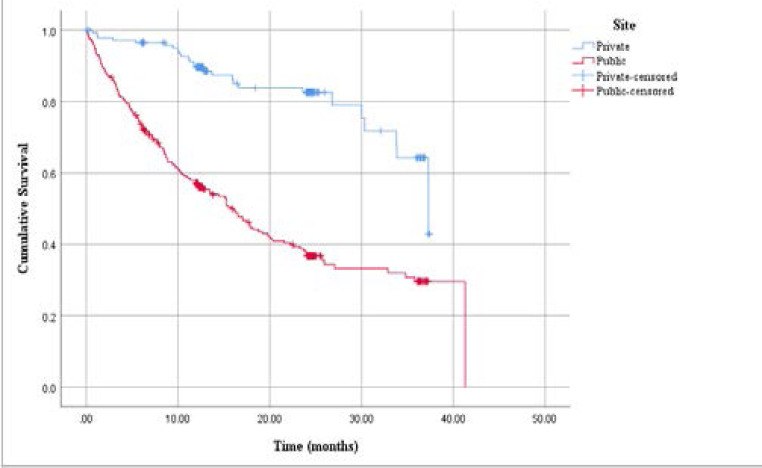
Differences in survival between the private and public sector cohorts

Table 4 summarises the survival rates and Figure 1 shows the Kaplan-Meier survival plot.

### Association between lifestyle factors and survival

For the combined cohort, there were weak, non-significant, inverse associations between survival and smoking (x2=2.34, Cramer's V=0.052, p=0.886) and between survival and alcohol consumption (x2=9.58, Cramer's V=0.086, p=0.386).

There was a non-significant, weak positive association between the frequency of achievement of vigorous-intensity weekly minutes and survival (x2=2.542, Cramer's V=0.090, p=0.468). There was a small-strength effect of the number of weekly vigorous-intensity minutes achieved on survival in patients with colorectal cancer (η=0.222, η2=0.049).

There was a medium-strength effect of BMI on survival (η=0.325, η2=0.106) and a large-strength effect of waist circumference on survival (η=0.0.516, η2=0.266).

## Discussion

To our knowledge, this is the first paper describing clinical profiles, lifestyle-related risk factors, and survival of an urban South African cohort with colorectal cancer. In this cohort, patients in the public sector were younger and mostly Black-African, whereas those in the private cohort were older and more frequently Caucasian. In concordance with our findings, previous epidemiological studies have shown Black individuals develop colorectal cancer earlier than Caucasian individuals^39^. Although there may be genetic and cultural factors, the differences in cancer incidence may be linked to socioeconomic status^40^.

The AJCC scores presented in the results showed a significant difference in cancer staging between those presenting in the private and public sectors. A potential explanation for this might be patient-related or linked to barriers to accessing appropriate care. Healthcare in South Africa is comprised of public and private sectors with vast differences separating the two. The public healthcare system serves approximately 80 percent of the South African population; with limited capacity to manage complicated conditions like colorectal cancer, which often requires highly specialised services in multiple disciplines^41^. The difference in cancer staging may in turn also explain the significant difference in survival between the two cohorts. Authors of an American study who analysed data collected from 1981 to 2013 showed younger age and the African race were significant risk factors for advanced staging of colorectal cancer^42^.

Evidence suggests that moderate-intensity exercise may have a biological effect in reducing colorectal cancer risk^43^. Patients undergoing cancer treatments may find non-vigorous intensity exercise more tolerable than vigorous-intensity exercise. Examples of moderate-intensity physical activity include brisk walking and dancing whereas running and aerobics are classified as vigorous-intensity physical activity^33^. Due to missing data, we only had access to the vigorous-intensity weekly exercise achieved by the patients in this cohort. Research to date has shown that vigorous-intensity exercise is not associated with improved survival outcomes^21^,^22^. Our findings support this, as we found only a small-strength effect of the vigorous-intensity weekly minutes achieved on survival. Unfortunately without data for moderate-intensity exercise, conclusions are difficult to make on patients' physical activity profiles.

Our results showed that BMI and waist circumference had moderate-and-large-strength effects respectively on survival. These findings support research which suggests that patients with increased BMI have better survival outcomes^7^,^14^,^18^. Patients with advanced-stage colorectal cancer experience weight loss, sarcopenia, and cachexia which may have a greater impct on patients who have a low BMI^14^. Therefore, being overweight may be protective in patients with advanced colorectal cancer^14^. Screening for ideal waist circumference and BMI may be essential in managing patients undergoing various treatments for colorectal cancer. Waist circumference is a simple, inexpensive measure used to determine central adiposity in men and women^44^. However, waist circumference cut-off points for determining colorectal cancer risk and survival are yet to be established in sub-Saharan Africa.

Physiotherapists can provide non-pharmacologic interventions to assist patients with their physical health needs during cancer treatments and pre-and-post-operatively45. As rehabilitation specialists, they are well trained to manage modifiable risk factors such as physical inactivity and can offer education on health behaviours like smoking, alcohol consumption, and basic nutritional practices46. Furthermore, physiotherapists have the resources and clinical reasoning to screen and refer patients to other healthcare providers as needed^45^. The data presented in this paper highlight the need for anthropometric screening and lifestyle education in this cohort presenting with colorectal cancer. Management of modifiable risk factors could influence colorectal cancer incidence and outcomes in South Africa.

Future research is required to determine the incidence of sarcopenia in South African patients with colorectal cancer and to determine its association with survival. This will assist us to re-define and streamline the role of physiotherapists in the management of these patients.

## Limitations to the study

Certain variables were considered for inclusion into the REDCap database after the start of data collection. This resulted in missing data for variables such as the ECOG score. Missing data was a limitation in calculating GPAQ scores.

The between-group differences demonstrated in the results of this study should be interpreted with caution due to the large difference in sample size between the two groups.

This cohort only represents a sample of the South African urban population of patients with colorectal cancer. Patients living in rural areas may present with a different profile. This could be due to several factors including access to healthcare which may affect the staging of cancer on the first presentation as well as the comorbidities diagnosed. Another limitation is that the cohort in this study is not a population-based sample.

## Conclusion

In this urban South African cohort, lifestyle factors known to be associated with colorectal cancer risk were found. Patients in the private sector cohort were older, had earlier stages of cancer staging, and had a higher percentage of current alcohol consumption when compared to the public cohort. There were significantly higher survival rates in the private sector cohort in comparison to the public sector cohort. Waist circumference and BMI were shown to have large-and-medium-strength positive effects respectively on survival.

Emphasis should be placed on screening for anthropometric data and education on maintaining an ideal waist circumference and BMI should be prioritised. As established healthcare professionals, physiotherapists are well-positioned to provide such screening and education to effect long-term lifestyle behaviour change and improve outcomes.
